# Restoration of mitochondrial dynamics protects against neurodegeneration in a mouse model of Alzheimer’s Disease

**DOI:** 10.1002/alz70861_108430

**Published:** 2025-12-23

**Authors:** Fanpeng Zhao

**Affiliations:** ^1^ Case Western Reserve University, Cleveland, OH USA

## Abstract

**Background:**

Mitochondrial dysfunction is a major pathological event of neurodegeneration including Alzheimer’s disease (AD). Expanding evidence demonstrate that an altered balance in mitochondrial dynamics is an important mechanism leading to mitochondrial and neuronal dysfunction during neurodegeneration. Thus, it is of great interest to test if restoration of mitochondrial dynamics has beneficial role in AD treatment.

**Method:**

5xFAD mouse model of AD was used to test whether MFN2 OE or MFN2 activator rescues mitochondrial dysfunctions and neurodegeneration. Behavioral tests, mitochondrial analysis, electrophysiological study, RNA‐seq and biochemical analysis were applied to examine the brain pathology in the AD mouse model.

**Result:**

In this study, we observed fragmented and damaged mitochondria in 5xFAD mouse brains, along with impaired mitochondrial respiration of synaptosome at 6 months. Restoration of mitochondrial dynamics by conditional overexpression (OE) of mitofusin‐2 (MFN2) in forebrain neurons rescues mitochondrial fragmentation and dysfunction in 5xFAD mouse brains. Importantly, MFN2 OE protects against long‐term potential reduction, synaptic loss, oxidative stress damage, amyloid plaque size, neuroinflammation and cognitive deficits in 5xFAD mice at 6 months. Amyloid plaque burden and neuronal death in plaque‐enriched layer V cortical regions can be alleviated in 5xFAD mice by MFN2 OE at 10 months. RNA sequencing (RNA‐seq) reveals disturbed transcriptomic profiles including increased inflammatory responses in 5xFAD mice, which is reversed by MFN2 OE. Specifically, MFN2 OE inhibits microglial activation, proinflammatory cytokine production, and NLRP3 inflammasome activation in 5xFAD mice. Lastly, intraperitoneal injection of compound BAY2402234, a brain penetrant MFN2 activator, rescues mitochondrial fragmentation, oxidative damage, and memory deficits in 5xFAD mice.

**Conclusion:**

Overall, our study demonstrated that the restoration of mitochondrial dynamics by inhibition of mitochondrial fragmentation can protect against neurodegeneration in 5xFAD mouse model and mitochondrial dynamic could be a promising therapeutic target for AD.

